# Taxes and front-of-package labels improve the healthiness of beverage and snack purchases: a randomized experimental marketplace

**DOI:** 10.1186/s12966-019-0799-0

**Published:** 2019-05-21

**Authors:** Rachel B. Acton, Amanda C. Jones, Sharon I. Kirkpatrick, Christina A. Roberto, David Hammond

**Affiliations:** 1 School of Public Health and Health Systems, University of Waterloo, 200 University Ave W, Waterloo, ON N2L 3G1 Canada; 2 Department of Public Health, University of Otago, Wellington, 23A Mein St., Newtown, Wellington, 6021 New Zealand; 3 Department of Medical Ethics and Health Policy, Perelman School of Medicine, University of Pennsylvania, 423 Guardian Drive, Philadelphia, PA 19104 USA

**Keywords:** Front-of-package labels, Health warnings, Taxes, Sugar tax, Experimental marketplace, Sugar-sweetened beverages

## Abstract

**Background:**

Sugar taxes and front-of-package (FOP) nutrition labelling systems are strategies to address diet-related non-communicable diseases. However, there is relatively little experimental data on how these strategies influence consumer behavior and how they may interact. This study examined the relative impact of different sugar taxes and FOP labelling systems on beverage and snack food purchases.

**Methods:**

A total of 3584 Canadians 13 years and older participated in an experimental marketplace study using a 5 (FOP label condition) × 8 (tax condition) between-within group experiment. Participants received $5 and were presented with images of 20 beverages and 20 snack foods available for purchase. Participants were randomized to one of five FOP label conditions (*no label*; *‘high in’ warning*; *multiple traffic light*; *health star rating*; *nutrition grade*) and completed eight within-subject purchasing tasks with different taxation conditions (beverages: *no tax*, *20% tax on sugar-sweetened beverages (SSBs)*, *20% tax on sugary drinks*, *tiered tax on SSBs*, *tiered tax on sugary drinks*; snack foods: *no tax*, *20% tax on high-sugar foods*, *tiered tax on high-sugar foods*). Upon conclusion, one of eight selections was randomly chosen for purchase, and participants received the product and any change.

**Results:**

Compared to those who saw no FOP label, participants who viewed the ‘high in’ symbol purchased less sugar (− 2.5 g), saturated fat (− 0.09 g), and calories (− 12.6 kcal) in the beverage purchasing tasks, and less sodium (− 13.5 mg) and calories (− 8.9 kcal) in the food tasks. All taxes resulted in substantial reductions in mean sugars (− 1.4 to − 4.7 g) and calories (− 5.3 to − 19.8 kcal) purchased, and in some cases, reductions in sodium (− 2.5 to − 6.6 mg) and saturated fat (− 0.03 to − 0.08 g). Taxes that included 100% fruit juice (‘sugary drink’ taxes) produced greater reductions in sugars and calories than those that did not.

**Conclusions:**

This study expands the evidence indicating the effectiveness of sugar taxation and FOP labelling strategies in promoting healthy food and beverage choices. The results emphasize the importance of applying taxes to 100% fruit juice to maximize policy impact, and suggest that nutrient-specific FOP ‘high in’ labels may be more effective than other common labelling systems at reducing consumption of targeted nutrients.

**Electronic supplementary material:**

The online version of this article (10.1186/s12966-019-0799-0) contains supplementary material, which is available to authorized users.

## Background

Diet-related non-communicable diseases are among the leading causes of premature death and disability worldwide [1]. Diets high in processed foods and low in fruits, vegetables and whole grains remain dominant in developed countries, and are supplanting more traditional diets in lower income countries [2, 3]. Several strategies have emerged to improve dietary intake at a population level, including the use of fiscal measures and front-of-package (FOP) nutrition labelling [4, 5].

Food and beverage taxes aim to increase the price of less healthy food and beverage products. Although some jurisdictions have applied health-oriented taxes to foods—such as those high in calories, sugars, sodium, or saturated and trans fats [6]—most have focused on beverages high in sugars, which are typically defined one of two ways [7]. Sugar-sweetened beverages (SSBs) are beverages containing ‘added sugar’ (any sugars added during processing or preparation [8]), such as regular soft drinks, sports drinks, flavoured waters, and fruit drinks [9]. In contrast, sugary drinks are defined based on the World Health Organization (WHO) criteria for ‘free sugars’ (i.e., all added sugars, plus those naturally present in honey, syrups, fruit juices, and fruit juice concentrates [7]), and therefore include all beverages under the umbrella of SSBs, plus 100% juice products. This study presented in this manuscript compares policies that target SSBs versus those that target the broader definition of sugary drinks.

To date, the vast majority of beverage taxes have been applied to SSBs. Mexico, UK, Ireland, France, South Africa, and Chile, as well as several US cities (e.g., Berkeley, Philadelphia, Boulder, Seattle) have all implemented SSB taxes [6, 10–17]. Evidence from experimental studies, observational assessments of real-world taxes, and simulation modelling suggests SSB taxes applied at a rate equivalent to at least 20% of a products’ price are likely to be an effective means of reducing purchasing and consumption of high-sugar beverages, as well as a strong incentive for product reformulation [18–25]. However, given their relative novelty, the optimal design of SSB taxes to reduce SSB consumption and encourage product reformulation while also generating revenue for investment in other health promotion efforts remains unclear. For example, the range of beverages subject to taxation varies considerably across jurisdictions: several exclude sugar-sweetened milks, some include diet beverages, and the vast majority exclude 100% fruit juice. Additionally, policies vary in the type of tax (e.g., excise, sales). Excise taxes apply price increases at the point of the manufacture, sale, or distribution of a good, whereas sales taxes are levied at the point of purchase [26]. Under the umbrella of excise taxes, the most common in the context of SSB taxes, price increases may be applied in a ‘specific’ format—either based on product volume or nutrient volume—or in an ‘*ad valorem*’ format, applied as a percentage of the product’s price (e.g., 20%) [26]. Some research suggests a specific excise tax based on beverage volume or sugars content may be preferable to a sales tax or *ad valorem* excise tax—both of which constitute a percentage price increase. Specific taxes create a higher relative price increase in cheaper goods, reducing the potential for consumers to choose less costly but equally unhealthy items [25–27]. Another emerging tax model is a tiered tax, which is a specific tax that applies varying price increases to products based on two or more predefined levels of sugar content or product volume. The UK’s excise beverage tax uses this tiered model based on beverage sugar content [14], while Mexico’s SSB regulations assign a specific excise tax, roughly equivalent to 1 cent per ounce of beverage [10]. To the authors’ knowledge, no experimental studies have directly compared the effectiveness of sugary drink taxes based on product volume (i.e., *ad valorem* excise) versus those based on sugar content (i.e., tiered) on consumer purchasing and consumption, and few have compared taxes that define SSBs in different ways.

FOP nutrition labels are another policy measure to promote healthy eating. FOP labelling systems seek to provide simple, interpretive information on the front of packaged food and beverage products to help consumers quickly and easily evaluate their healthfulness [28]. An increasing variety of these labelling systems are being implemented internationally [28]. FOP labelling systems can broadly be categorized as ‘nutrient-specific’ systems that provide information on one or more specific nutrients (e.g., Chile’s ‘high in’ nutrient warnings, UK’s traffic light labels) or ‘summary indicator’ systems that provide a score or rating of the overall nutrient profile of a product (e.g., Australia and New Zealand’s Health Star Rating, France’s five-colour Nutri-Score) [28]. Reviews of the existing evidence suggest that FOP nutrition labels may be an effective approach to help consumers choose healthier products; however, there is no consensus as to which FOP label system may be most effective [29–32]. Further, a majority of existing research has focused on the first generation of FOP labelling systems, such as star ratings, traffic light symbols, and guideline daily amount labels. There is less evidence on more recent FOP systems such as ‘high in’ warning labels and France’s five-colour Nutri-Score system.

Canada is currently finalizing regulations for a mandatory nutrient-specific FOP labelling system. Similar to Chile’s system, the new policy will require all packaged foods and beverages to display a ‘high in’ symbol if they exceed thresholds for sugars, sodium, or saturated fats [33]. In addition, health advocacy groups are increasingly calling for a national sugary drink tax in Canada [34, 35]. There is a need for evidence comparing the relative effectiveness of different taxation strategies and FOP labelling formats—as well as how these policy measures interact when applied in combination—to help inform the implementation of FOP labelling and tax policies in Canada and other countries. Additionally, it is unknown whether policies have similar impacts on purchasing and consumption of foods compared to beverages.

The current study, which utilized an experimental marketplace, sought to test the relative impact of (1) different food and beverage sugar taxes, and (2) different formats of nutrient-specific and summary indicator FOP nutrition labels on Canadian consumers’ purchasing of sugars, sodium, saturated fats, and calories. Purchases were assessed using a range of beverage and snack food products typically available at a convenience or corner store, which provided a wide range of nutrient profiles. The study examined five primary research questions: (1) Does a tax on SSBs impact purchases of sugars, sodium, saturated fats and calories differently than a tax on sugary drinks?; (2) Does a tiered specific excise tax based on sugar content impact purchases differently than an *ad valorem* tax?; (3) Do nutrient-specific FOP nutrition labels (e.g., ‘high in’ warnings, multiple traffic lights) impact purchases differently than summary indicator FOP systems (e.g., health star ratings, 5-colour nutrition scores)?; (4) Do sugar taxes and FOP labels have similar impacts on purchases when applied to foods compared to beverages?; and (5) Do the effects of sugar taxes and FOP labelling systems interact when applied in combination?

## Methods

### Study design

The study was conducted from March to May 2018. Ethical approval was granted by the Office of Research Ethics at the University of Waterloo (ORE #22494).

An experimental marketplace is an approach commonly used in the field of behavioural economics and marketing to study actual consumer behaviour, and provides the opportunity to manipulate price and other variables of interest to assess their influence on consumers’ purchases [36, 37]. Participants are provided with a sum of money, and presented with multiple products available for purchase. If the participant does not spend the entire sum of money, they are permitted to keep the remainder, along with the product they selected. In this way, participants spend real money and incur a financial cost for their purchases, leading to more realistic product selections [36, 37].

### Study protocol

#### Participants and recruitment

Participants aged 13 years and older were recruited using convenience sampling from large shopping centres in three Canadian cities (Kitchener, Waterloo, and Toronto) within the province of Ontario. Youth are an important subpopulation to include in diet-related research due to their higher consumption of nutrients of concern and differed interactions with tax and labelling policies compared to older populations [38–41]. Research assistants were stationed at booths in high-traffic areas in the shopping centres, and approached potential participants to ask if they were interested in participating in a study on food and beverage purchasing patterns. All interested participants were asked to provide their age prior to giving written informed consent and beginning the study. Additional written informed consent from a parent or guardian was required for all participants under 16 years; if a parent or guardian was not present, the shopper was not permitted to participate. Participants completed the study at the booth with the research assistant, immediately following consent.

#### Purchasing tasks

**Fig. 1:**
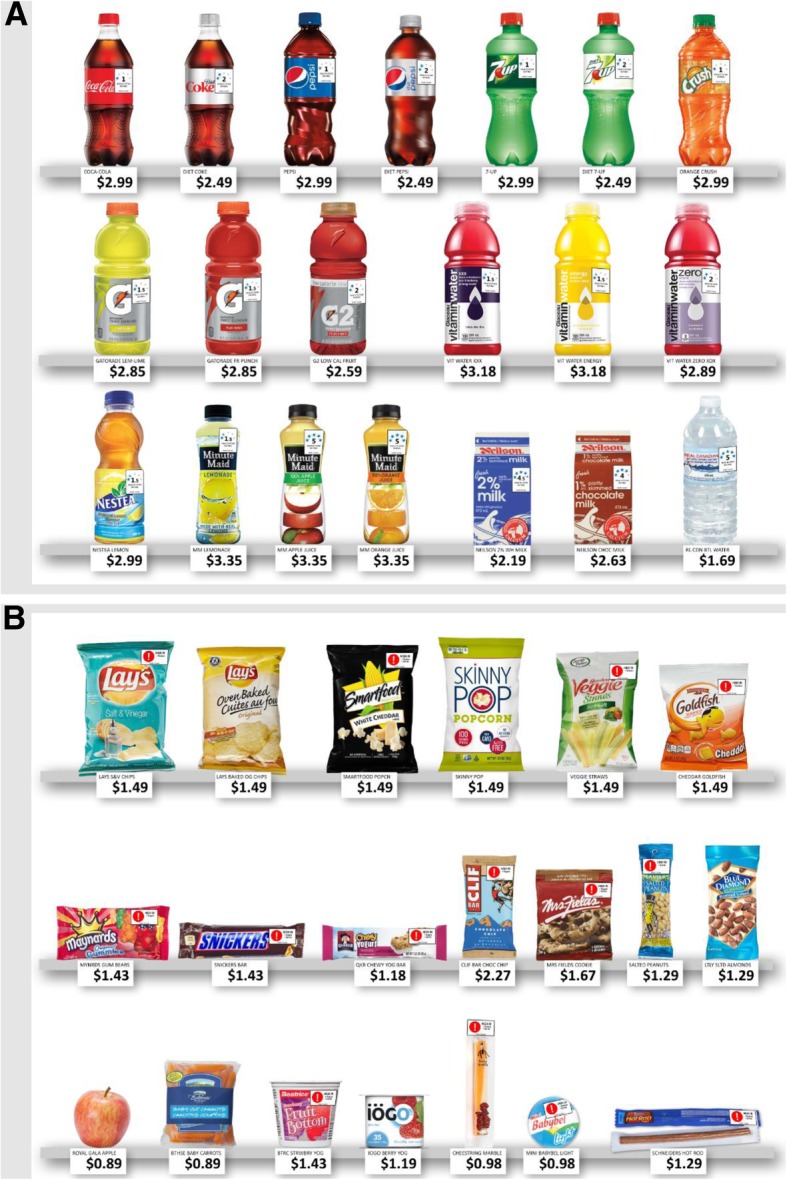
Example product shelf images showing two combinations of FOP and taxation conditions: **a** beverages with *health star rating* labels and *tiered SD* tax, **b** foods with *high in* labels and *20%* sugar tax

The experimental purchasing tasks were delivered in the format of a 5 (FOP label condition) × 8 (tax condition) between-within group experiment. A visual depiction of the purchasing task protocol is available in Additional file 1 (Figure S1). Participants were randomly assigned to one of five FOP label conditions. Within their assigned label condition, participants completed eight consecutive purchasing tasks, which each corresponded to a different tax condition. In each of the eight purchasing tasks, participants were shown a selection of beverage or snack products on a large (62.5 × 50 cm) laminated print-out, which was designed to replicate the appearance of a grocery or convenience store shelf (Fig. 1). A new print-out was shown for each purchasing task, reflecting the appropriate label and tax condition for that purchase. In the first five purchases, participants selected from 20 different beverage products. In the last three purchases, participants selected from 20 different snack food products. The order of the tax conditions was randomized within the five beverage tasks and within the three food tasks. At the end of the survey, the program randomly selected one of the eight purchasing tasks to be the actual purchase, and the participant received the product selected with that task.

Prior to each of the eight purchasing tasks, research assistants emphasized the following points to each participant: (1) they had a budget of $5.00 to purchase one item, (2) the labels may be different from what they’ve seen in the past, (3) the prices may have changed since the last task, and (4) they would receive their change from the $5.00 and the actual food or beverage product from one of the eight purchases. Research assistants were instructed to not engage in discussion or answer questions about nutrition, diet, or food policies. For each task, participants made their selection on an iPad after viewing the large shelf image. Participants did not know which purchase selection they would receive (along with any change from the $5.00) until the end of the experiment and were instructed to treat all eight tasks as real purchases.

Upon completion of the eight purchasing tasks, each participant was asked “In all of the previous purchasing tasks, did you notice any nutrition labels or symbols on the front of the food and beverage packages?”, with response options “yes”, “no”, “don’t know”, or “refuse to answer”.

#### Experimental conditions

**Fig. 2:**
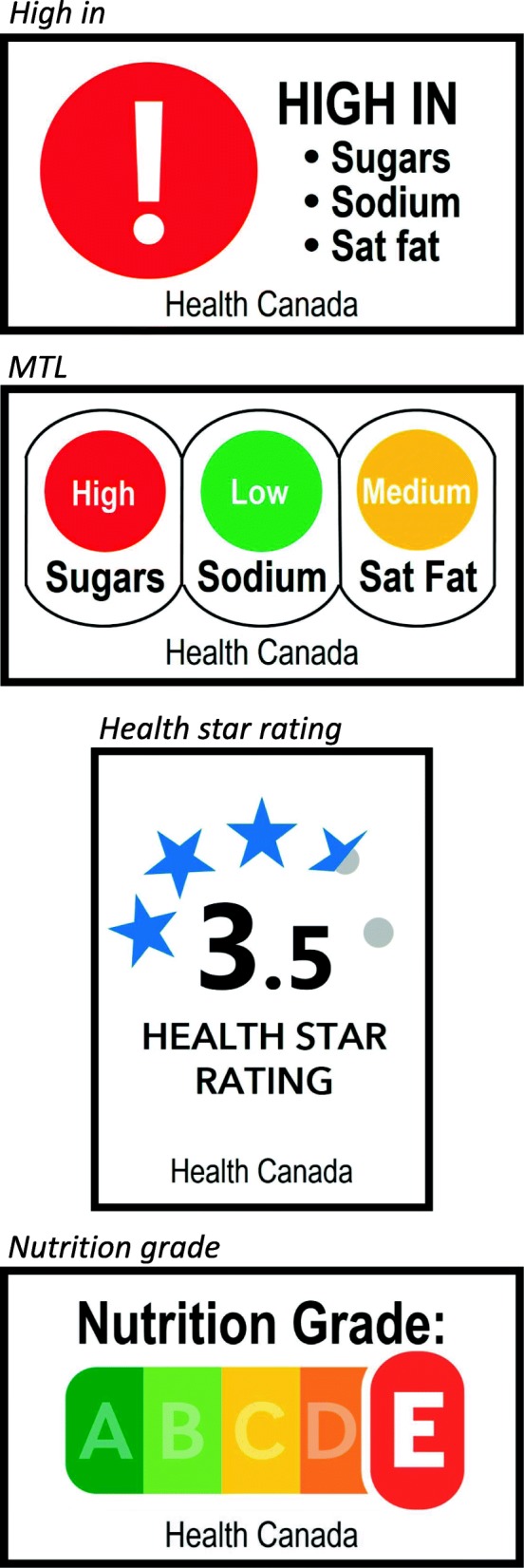
Images of label conditions, excluding *no label* (control). From top to bottom: *high in*, *MTL*, *health star rating*, and *nutrition grade*

Five FOP label conditions were tested, including two nutrient-specific labels and two summary indicator systems. The FOP label conditions were *no label* (control); a *high in* warning system labelling foods high in sugars, sodium or saturated fats; a multiple traffic light system (*MTL*) for sugars, sodium and saturated fats; a *health star rating* label; and a five-colour *nutrition grade* label (Fig. 2).

The *high in* warning system was modelled after early iterations of Health Canada’s proposed FOP warning symbols for foods high in sugars, sodium and saturated fats, with nutrient thresholds based on Health Canada’s proposed guidelines [33]. The *MTL* system was loosely based on the UK’s voluntary traffic light labelling system [42]. To ensure comparability with the *high in* system, MTL labels were displayed only for sugars, sodium and saturated fats. Criteria for ‘high’, ‘medium’ and ‘low’ were based on the UK’s regulations [42]; however, in two cases in which the *MTL* was incongruent with the *high in* warning labels, the *MTL* was adjusted to match Health Canada *high in* warnings. The *health star rating* label design and scoring system were modeled after Australia and New Zealand’s Health Star Rating system [43]. The *nutrition grade* system was designed based on France’s Nutri-Score system [44]. Due to differences in criteria and scoring algorithms across the two summary indicator systems, the *nutrition grade* scores were adjusted to match those of the *health star rating* for the purposes of this study (i.e., 0.5 to 1 stars = ‘E’ nutrition grade; 1.5 to 2 stars = ‘D’; 2.5 to 3 stars = ‘C’; 3.5 to 4 stars = ‘B’; 4.5 to 5 stars = ‘A’). The FOP labels were not applied to fresh fruits or vegetables (i.e., the apple and carrots) to align with most real-world FOP nutrition labelling systems. See Additional file 1: Table S1 for details on the FOP labels assigned to all food and beverage products.

Five beverage-based sugar tax conditions (Table 1) were tested: *no tax* (control), a 20% *ad valorem* tax on SSBs (*20% SSB*), a 20% *ad valorem* tax on sugary drinks (*20% SD*), a tiered specific tax on SSBs (*tiered SSB*), and a tiered specific tax on sugary drinks (*tiered SD*). Beverages were categorized as SSBs if they contained added sugar, as previously defined [8]. Beverages were categorized as sugary drinks if they contained free sugar, as defined by WHO [7]. *20% SSB* and *20% SD* taxes were applied to beverages containing more than 5 g of added or free sugars (respectively) per 100 ml. *Tiered SSB* and *tiered SD* taxes applied a 10% price increase to beverages containing 5 to 8 g, or a 20% price increase to beverages containing more than 8 g of added or free sugars per 100 ml (modelled after the SSB tax implemented in the UK [45]). The study also tested three food-based sugar tax conditions: *no tax* (control), a 20% *ad valorem* tax on high-sugar foods (*20%*), and a tiered specific tax on high-sugar foods (*tiered*). Here, the *20%* tax was assigned to all foods containing more than 10 g of total sugars per 100 g; the *tiered* tax applied a 10% price increase to foods containing more than 10 to 20 g of total sugars per 100 g, and a 20% price increase to foods containing more than 20 g of total sugars per 100 g. The SSB and SD tax formats were not applicable to the snack food purchases. Additional file 1 provides details on how the taxes were assigned to each product (Table S2), as well as nutrition information of all products (Table S3).

**Table 1:** Summary of sugar tax conditions

Beverage purchases	
1	No tax (control)
2	20% SSB
3	20% SD
4	Tiered SSB
5	Tiered SD
Food purchases	
6	No tax (control)
7	20%
8	Tiered

*SSB* sugar-sweetened beverage, *SD* sugary drink

#### Sociodemographic measures

Following the purchasing tasks and using the iPad, participants provided information on their previous 7-day sugary drink consumption using a brief single-item beverage frequency measure (“During the past 7 days, how many sugary drinks did you have?”) [46]. Participants also reported their age, sex, ethnicity, education, income adequacy (“Thinking about your total monthly income, how difficult or easy is it for you to make ends meet?”), and height and weight. Self-reported height and weight were used to calculated body mass index (BMI), which was categorized into “underweight”, “normal weight”, “overweight” and “obese” using the WHO thresholds [47]. BMIs for participants 19 years of age or younger were calculated using growth charts as recommended by CDC and WHO guidelines [48, 49]. All survey items were completed after the experiment to minimize influence on participants’ behaviours in the purchasing tasks.

#### Remuneration

After participants had completed all survey items, the survey program randomly selected one of their eight purchasing tasks. Research assistants gave participants their actual food or beverage product and their change from the $5.00 corresponding to that purchase.

### Outcome variables

Four primary outcomes were explored: grams of sugars purchased, milligrams of sodium purchased, grams of saturated fats purchased, and number of calories purchased per task. All four outcomes were measured based on the total amount of sugars, sodium, saturated fats, or calories in the entire package of the product selected in each purchasing task; all products were single-serving sized and expected to be consumed in one sitting. All four nutrient outcomes were assessed for both foods and beverages. Although sugars and calories were the principal nutrients of concern for the beverages, several presented beverages contained substantial amounts of sodium (i.e., sports drinks) and saturated fat (i.e., milks). The impacts of the sugar-based taxes on purchasing were explored for all four nutrient outcomes (including sodium and saturated fats) so as to capture any potential ‘spillover’ effects of sugar-based taxes [50]. Secondary outcomes included potential interaction effects between FOP labelling and taxes, as well as participants’ reported noticing of the FOP nutrition labels.

### Analyses

Chi square tests (for categorical variables) and one-way ANOVAs (for linear variables) were used to test for sociodemographic differences between experimental conditions (FOP label format). Separate two-tailed repeated-measures ANOVAs were used to investigate the effects of labelling and tax on the amount of sugars, sodium, saturated fats, and calories purchased; foods and beverage purchases were analysed separately, resulting in a total of eight ANOVAs. Repeated-measures ANOVAs were used to account for the repeated nature of the purchasing tasks. All ANOVAs included a *tax condition* × *label condition* interaction. In the case that an ANOVA violated the assumption of sphericity [51], Greenhouse-Geisser corrections [52] were applied to the results. All statistical analyses were conducted using SPSS software (version 25.0; IBM Corp., Armonk, NY; 2017). The significance threshold was set at 0.05 for all tests. No adjustments for multiple comparisons were applied. It has been suggested that experiments based on distinct, conceptually sound a priori hypotheses and which have discrete, separate experimental arms should not apply adjustments for multiple comparisons [53–55]. Results should be interpreted by the strength and magnitude of the effect sizes, *p*-values, and confidence intervals.

## Results

Sample characteristics are presented in Table 2. A total of 3702 participants (96.7% of those who consented) completed the study; 118 participants were removed due to data quality concerns reported by the research assistants (e.g., significant cognitive difficulties or distraction, visual impairment, substantial influence from peers), resulting in a final sample size of 3584. Participants spent an average of 17.3 min to complete the purchasing tasks and subsequent survey items.

**Table 2:** Sociodemographic characteristics of sample (*N* = 3584) and test results for differences across conditions

Characteristic	%	Test for differences
City		χ^2^ = 5.7 (*p* = .684)
Kitchener	17.5	
Toronto	41.2	
Waterloo	41.4	
Age (years)		χ^2^ = 25.8 (*p* = .058)
13–18	15.3	
19–25	31.0	
26–35	20.6	
36–45	11.9	
> 45	21.3	
Gender		χ^2^ = 0.8 (*p* = .940)
Male	44.0	
Female	56.0	
Weekly beverage frequency		*F* = 1.0 (*p* = .404)
Number of sugary drinks (*mean*)	4.0	
Ethnicity		χ^2^ = 7.3 (*p* = .839)
White	44.9	
Other/mixed	50.3	
Indigenous	3.3	
Not stated	1.6	
Education		χ^2^ = 1.9 (*p* = .985)
High school or less	26.6	
CEGEP/Trade School/College (partial or complete)	11.7	
University (partial or complete)	61.7	
Income adequacy		χ^2^ = 8.2 (*p* = .416)
‘Very difficult’ or ‘Difficult’	19.5	
‘Neither easy nor difficult’	41.4	
‘Easy’ or ‘Very easy’	39.1	
BMI classification		χ^2^ = 12.3 (*p* = .726)
Underweight	3.3	
Normal weight	46.0	
Overweight	22.8	
Obese	12.1	
Not reported	15.8	

*CEGEP* Collège d’enseignement général et professionnel (general and vocational college); BMI, body mass index

There were no significant differences in sociodemographic measures across the between-group (FOP label format) experimental conditions (Table 2).

### Label noticing

Among participants who were assigned to view products with a FOP label, 51.5% reported noticing any nutrition labels or symbols on the food and beverage packages. Table 3 presents the proportion of participants who reported noticing nutrition labels or symbols across each label condition.

**Table 3:** Participant responses to “In all of the previous purchasing tasks, did you notice any nutrition labels or symbols on the front of the food and beverage packages?”, by label condition (*N* = 3584)

	*Label Condition*				
*Response*	No FOP label (control) %	High in %	MTL %	Health star rating %	Nutrition grade %
	*n* = 726	*n* = 714	*n* = 709	*n* = 718	*n* = 717
Yes	28.4	58.3	45.0	52.5	50.3
No	71.2	40.3	53.7	46.0	48.1
Don’t know	0.4	1.4	1.3	1.5	1.5

*FOP* front-of-package, *MTL* multiple traffic light

### Beverage purchasing tasks

**Fig. 3:**
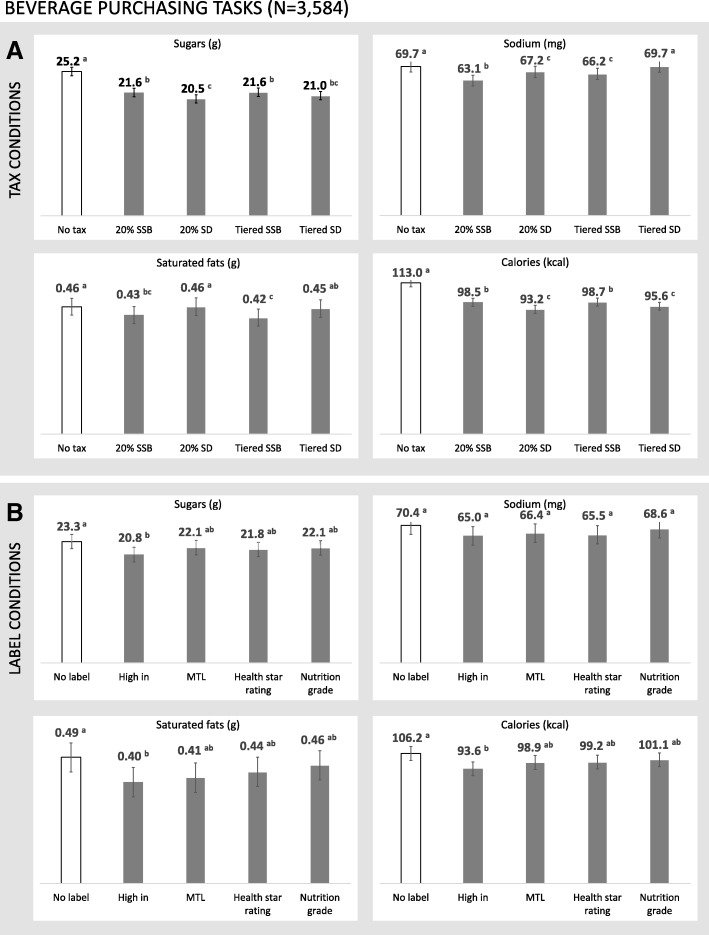
Sugars, sodium, saturated fats, and calories in purchased beverages within an experimental marketplace in which (**a**) tax conditions and (**b**) FOP label conditions varied. Error bars represent 95% confidence intervals for the mean estimates. ^a,b,c^ Values with differing superscript letters indicate tests for which *p* < .05 in a repeated-measures ANOVA

Mean amounts of sugars, sodium, saturated fats and calories purchased in the beverage tasks are presented in Fig. 3. Repeated-measures ANOVA results are presented in Table 4, including pairwise comparisons between all tax and labelling conditions. There were no significant two-way interactions between tax and labelling condition for any of the four outcomes in the beverage tasks.

**Table 4:** Repeated-measures ANOVA results for sugars, sodium, saturated fats, and calories in beverage and food purchase selections within an experimental marketplace with varied tax and FOP label conditions (*N* = 3584)

	Sugars	Sodium	Saturated fats	Calories
BEVERAGE PURCHASES				
*Main Effects Model Statistics*				
Tax condition	*F* (3.95, 14126.96) = 68.55^*^	*F* (3.98, 14233.92) = 10.01^*^	*F* (3.97, 14219.73) = 3.45^*^	*F* (3.96, 14158.14) = 71.78^*^
Label condition	*F* (4, 3579) = 1.63	*F* (4, 3579) = 0.89	*F* (4, 3579) = 1.74	*F* (4, 3579) = 2.50^*^
Tax condition × Label condition	*F* (15.79, 14126.96) = 0.45	*F* (15.91, 14233.92) = 1.23	*F* (15.89, 14219.73) = 1.23	*F* (15.82, 14158.14) = 0.44
*Pairwise comparisons: Tax conditions*	*Mean difference* g (95% CI)	*Mean difference* mg (95% CI)	*Mean difference* g (95% CI)	*Mean difference* kcal (95% CI)
no tax – 20% SSB	3.68 (3.02, 4.34)^*^	6.62 (4.27, 8.97)^*^	0.03 (0.01, 0.06)^*^	14.47 (11.84, 17.09)^*^
no tax – 20% SD	4.77 (4.10, 5.45)^*^	2.51 (0.04, 4.97)^*^	-0.001 (-0.03, 0.03)	19.78 (17.11, 22.46)^*^
no tax – tiered SSB	3.61 (2.97, 4.25)^*^	3.51 (1.08, 5.93)^*^	0.04 (0.01, 0.07)^*^	14.27 (11.73, 16.82)^*^
no tax – tiered SD	4.22 (3.56, 4.87)^*^	0.02 (-2.50, 2.54)	0.01 (-0.02, 0.03)	17.42 (14.81, 20.02)^*^
20% SSB – 20% SD	1.09 (0.48, 1.70)^*^	-4.11 (-6.45, -1.77)^*^	-0.03 (-0.06, -0.01)^*^	5.32 (2.82, 7.82)^*^
20% SSB – tiered SSB	-0.07 (-0.69, 0.55)	-3.12 (-5.50, -0.73)^*^	0.01 (-0.02, 0.04)	-0.19 (-2.66, 2.28)
20% SSB – tiered SD	0.54 (-0.08, 1.15)	-6.60 (-9.05, -4.15)^*^	-0.02 (-0.05, 0.01)	2.95 (0.46, 5.45)*
20% SD – tiered SSB	-1.16 (-1.77, -0.55)*	1.00 (-1.41, 3.40)	0.04 (0.01, 0.07)^*^	-5.51 (-7.99, -3.03)^*^
20% SD – tiered SD	-0.55 (-1.16, 0.05)	-2.49 (-4.88, -0.09)^*^	0.01 (-0.02, 0.03)	-2.36 (-4.75, 0.02)
tiered SSB – tiered SD	0.61 (-0.01, 1.22)	-3.48 (-5.89, -1.08)^*^	-0.03 (-0.06, -0.01)^*^	3.15 (0.69, 5.60)^*^
*Pairwise comparisons: Label conditions*	*Mean difference* g (95% CI)	*Mean difference* mg (95% CI)	*Mean difference* g (95% CI)	*Mean difference* kcal (95% CI)
no label – high in	2.50 (0.56, 4.45)^*^	5.35 (-1.27, 11.97)	0.10 (0.02, 0.18)^*^	12.62 (4.65, 20.59)^*^
no label – MTL	1.18 (-0.77, 3.13)	3.92 (-2.71, 10.55)	0.08 (-0.01, 0.16)	7.36 (-0.62, 15.34)
no label – health star rating	1.53 (-0.42, 3.47)	4.87 (-1.74, 11.48)	0.06 (-0.02, 0.14)	7.03 (-0.93, 14.98)
no label – nutrition grade	1.22 (-0.72, 3.16)	1.73 (-4.88, 8.35)	0.03 (-0.05, 0.11)	5.16 (-2.80, 13.12)
high in – MTL	-1.32 (-3.28, 0.64)	-1.43 (-8.09, 5.23)	-0.02 (-0.10, 0.06)	-5.26 (-13.28, 2.75)
high in – health star rating	-0.98 (-2.93, 0.98)	-0.48 (-7.12, 6.16)	-0.04 (-0.12, 0.04)	-5.59 (-13.58, 2.40)
high in – nutrition grade	-1.28 (-3.23, 0.67)	-3.62 (-10.25, 3.02)	-0.07 (-0.15, 0.02)	-7.47 (-15.34, 0.62)
MTL – health star rating	0.35 (-1.61, 2.30)	0.95 (-5.70, 7.60)	-0.02 (-0.10, 0.06)	-0.33 (-8.34, 7.67)
MTL – nutrition grade	0.04 (-1.92, 1.99)	-2.19 (-8.84, 4.47)	-0.05 (-0.13, 0.03)	-2.20 (-10.21, 5.80)
health star rating – nutrition grade	-0.31 (-2.26, 1.64)	-3.13 (-9.76, 3.50)	-0.03 (-0.11, 0.06)	-1.87 (-9.85, 6.11)
FOOD PURCHASES				
*Main Effects Model Statistics*				
Tax condition	*F* (1.99, 7125.23) = 50.02^*^	*F* (2, 7158) = 2.64	*F* (2.00, 7145.09) = 5.12^*^	*F* (2, 7158) = 15.04^*^
Label condition	*F* (4, 3579) = 0.28	*F* (4, 3579) = 2.24	*F* (4, 3579) = 1.48	*F* (4, 3579) = 2.87^*^
Tax condition × Label condition	*F* (7.96, 7125.23) = 0.84	*F* (8, 7158) = 0.61	*F* (7.99, 7145.09) = 1.19	*F* (8, 7158) = 0.99
*Pairwise comparisons: Tax conditions*	*Mean difference* g (95% CI)	*Mean difference* mg (95% CI)	*Mean difference* g (95% CI)	*Mean difference* kcal (95% CI)
no tax – 20%	1.54 (1.20, 1.88)^*^	-3.93 (-8.08, 0.22)	0.08 (0.03, 0.13)^*^	6.71 (4.20, 9.21)^*^
no tax – tiered	1.37 (1.04, 1.71)^*^	-4.42 (-8.59, -0.26)^*^	0.05 (0.01, 0.10)^*^	5.31 (2.77, 7.85)^*^
20% – tiered	-0.16 (-0.48, 0.16)	-0.49 (-4.58, 3.60)	-0.03 (-0.08, 0.02)	-1.40 (-3.94, 1.15)
*Pairwise comparisons: Label conditions*	*Mean difference* g (95% CI)	*Mean difference* mg (95% CI)	*Mean difference* g (95% CI)	*Mean difference* kcal (95% CI)
no label – high in	0.24 (-0.64, 1.12)	13.42 (0.78, 26.05)^*^	0.12 (-0.01, 0.26)	8.97 (1.10, 16.84)^*^
no label – MTL	-0.04 (-0.92, 0.85)	15.03 (2.37, 27.69)^*^	0.12 (-0.02, 0.25)	11.43 (3.55, 19.31)^*^
no label – health star rating	0.33 (-0.55, 1.21)	11.50 (-1.12, 24.12)	0.10 (-0.03, 0.23)	8.05 (0.19, 15.91)^*^
no label – nutrition grade	0.28 (-0.60, 1.16)	2.27 (-10.35, 14.89)	0.02 (-0.12, 0.15)	2.23 (-5.63, 10.09)
high in – MTL	-0.27 (-1.16, 0.61)	1.61 (-11.10, 14.32)	-0.004 (-0.14, 0.13)	2.46 (-5.46, 10.38)
high in – health star rating	0.09 (-0.79, 0.97)	-1.92 (-14.59, 10.75)	-0.02 (-0.16, 0.11)	-0.92 (-8.81, 6.97)
high in – nutrition grade	0.04 (-0.84, 0.92)	-11.15 (-23.82, 1.53)	-0.11 (-0.24, 0.03)	-6.74 (-14.63, 1.16)
MTL – health star rating	0.36 (-0.52, 1.25)	-3.53 (-16.22, 9.17)	-0.02 (-0.15, 0.12)	-3.38 (-11.28, 4.53)
MTL – nutrition grade	0.31 (-0.57, 1.20)	-12.76 (-25.45, -0.06)^*^	-0.10 (-0.24, 0.03)	-9.20 (-17.10, -1.29)^*^
health star rating – nutrition grade	-0.05 (-0.93, 0.83)	-9.23 (-21.89, 3.43)	-0.08 (-0.22, 0.05)	-5.82 (-13.70, 2.07)

95% CI, 95% confidence interval; SSB, sugar-sweetened beverage; SD, sugary drink; MTL, multiple traffic light

^*^p<.05

#### Taxes

Participants purchased fewer grams of sugars and calories in all tax conditions (*20% SSB*, *20% SD*, *tiered SSB*, *tiered SD*) compared to the *no tax* control condition (Table 4). The *20% SD tax* condition resulted in less sugars and calories purchased compared to the *20% SSB* and *tiered SSB* conditions. Participants purchased fewer calories in the *tiered SD* condition compared to the *20% SSB* and *tiered SSB* taxes.

For the *20% SSB*, *20% SD*, and *tiered SSB* tax conditions, participants’ beverage purchase selections contained less sodium compared to the *no tax* control condition. The *20% SSB* tax resulted in less sodium purchased in comparison to the *20% SD*, *tiered SSB*, and *tiered SD* tax conditions. The *20% SD* and *tiered SSB* conditions resulted in less sodium purchased compared to the *tiered SD* condition.

Participants purchased fewer grams of saturated fats in the *20% SSB* and *tiered SSB* tax conditions compared to the *no tax* control condition. The *20% SSB* tax condition also resulted in fewer grams of saturated fats purchased compared to the *20% SD* condition. Participants purchased fewer grams of saturated fats in the *tiered SSB* condition compared to the *20% SD* and *tiered SD* taxes.

#### FOP labelling

Participants assigned to the *high in* label condition purchased beverages containing less sugars, saturated fats, and calories compared to the *no label* control condition (Table 4). There were no significant differences in amount of sodium purchased between any of the labelling conditions in the beverage purchasing tasks.

### Food purchasing tasks

**Fig. 4:**
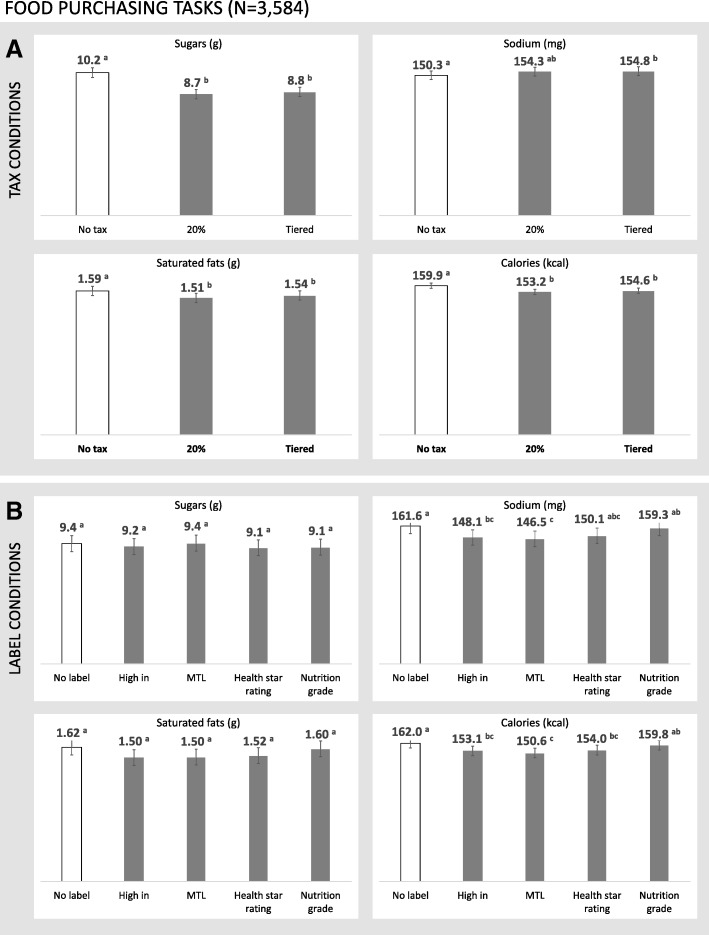
Sugars, sodium, saturated fats, and calories in purchased foods within an experimental marketplace in which (**a**) tax condition and (**b**) FOP label conditions varied. Error bars represent 95% confidence intervals for the mean estimates. ^a,b,c^ Values with differing superscript letters indicate tests for which *p* < .05 in a repeated-measures ANOVA

Mean grams of sugars, sodium, saturated fats, and calories purchased in the food purchasing tasks are presented in Fig. 4. Repeated-measures ANOVA results for the food tasks are presented in Table 4. There were no significant two-way interactions between tax and labelling condition for any of the four outcomes in the food tasks.

#### Taxes

Participants selected snack foods with less sugars, saturated fats, and calories in both the *20%* and *tiered* conditions compared to the *no tax* control. The *tiered* food tax resulted in a higher amount of sodium purchased in comparison to the control condition.

#### FOP labelling

There were no significant differences in the amount of sugars or saturated fats in the snack food purchase selections between any of the FOP labelling conditions. Participants assigned to the *high in* and *MTL* conditions purchased less sodium and fewer calories compared to the *no label* control condition, as did those assigned to the *MTL* compared to the *nutrition grade*. Participants who viewed the *health star rating* also purchased fewer calories than those in the *no label* control condition.

## Discussion

The findings suggest that sugar-based taxes and FOP nutrition labels can influence purchasing behaviour for beverage and snack purchases. As expected, the sugar-based taxes had the greatest impact on amounts of sugars and calories purchased. Within the beverage purchasing tasks, participants purchased products with up to 19% less sugars (− 4.7 g) and up to 18% fewer calories (− 19.8 kcal) compared to no tax. There were also substantial reductions in the foods purchased: sugar levels were 14 to 15% lower (− 1.4 to − 1.5 g) and calories were 3 to 4% lower (− 5.3 to − 6.7 g) under the tax conditions versus no tax. Although all tax formats for both beverages and foods affected the amounts of sugars and calories purchased, reductions were greatest when the tax was applied to 100% juice products in the ‘sugary drinks’ conditions as opposed to only sugar-sweetened beverages. Modelling studies suggest that including 100% juice in sugary drink taxes substantially increases the population-level health and economic impact of sugary taxes, mainly because fruit juice is one of the most frequently consumed sugary drinks in Canada and other Western countries [34, 56].

Although the taxes tested were based on sugar content, they also resulted in reductions in sodium and saturated fats purchased. For beverages, reductions in both sodium and saturated fats were as large as 9% (− 6.6 mg sodium; − 0.04 g saturated fat), and were driven mainly by switching away from sports drink and milk products, respectively. Similar reductions in saturated fat were observed among food purchases. As is the case in the broader food supply, the high-sugar foods presented in this study were often high in sodium and saturated fats as well [57], leading to ‘spillover’ effects of sugar taxes. However, participants purchased foods higher in sodium under the tax vs. no-tax conditions. These results suggest potential trade-off effects for snack foods: in order to avoid more expensive sugary foods, participants may have been more likely to switch to alternative snacks containing more sodium. To our knowledge, very little research has examined the compensatory effects of sugar taxes on purchases of other nutrients of concern such as sodium or saturated fats. Given an increasing focus on overall dietary patterns rather than isolated nutrients or foods [58], research with this expanded focus is an important contribution to the literature. The potential ‘spillover’ or compensatory effects of sugar taxes—whether positive or negative—should be key considerations for policymakers implementing sugar-based taxes.

Few differences were observed among taxes assigned based on product price (20% *ad valorem* tax conditions) and those assigned based on sugars content (tiered specific tax conditions). Although these tax structures may have similar impacts on consumer behaviour, they may have a different impact on industry behaviour, in terms of product reformulation. A tiered specific tax—based on either product volume or sugar content—may be more effective than a single-level *ad valorem* tax in motivating manufacturers to reduce sugar content, since tiered taxes offer intermediate sugar thresholds that may be easier to achieve [25]. Reports from the UK suggest that their tiered SSB tax has incentivized manufacturers to produce lower-sugar product formulations in efforts to avoid the levy [59]. Further research assessing the more novel tiered tax formats would be beneficial for policymakers considering a tax strategy.

For the FOP labels, the nutrient-specific *high in* warning performed most consistently in terms of reducing amounts of energy and the nutrients of interest. Participants in the *high in* condition purchased beverages with 11% less sugar (− 2.5 g), 18% less saturated fat (− 0.1 g), and 12% fewer calories (− 12.6 kcal) compared to the control condition. Similarly, in the food purchasing tasks, the *high in* warning produced an 8% reduction in sodium (− 13.5 mg) and a 5% reduction in calories (− 8.9 kcal) purchased. Although these reductions may appear modest at an individual level, they may translate to substantial reductions at a population level. The *MTL* and *health star rating* formats produced less consistent reductions in sodium and calories, while the *nutrition grade*—modelled after France’s Nutri-Score system—had minimal effects, resulting in similar outcomes to the control condition in all cases. Given the focus in this study on nutrient-specific outcomes, it is perhaps not surprising that the nutrient-specific FOP formats produced the greatest reductions in the targeted nutrients. It is also notable that ‘high in’ labels were most likely to be noticed compared to the other FOP labels, which highlights the importance of the general design and ‘salience’ of labels to engage consumers’ attention [60]. These results reflect similar findings from a range of experimental studies investigating nutrient-specific FOP warnings [61–66]. The poor performance of the five-colour nutrition grade in this study is in contrast to more promising results from France on the Nutri-Score system [67]; however, these differences may be due to the focus of the current study’s outcomes on specific nutrients of concern rather than overall nutritional quality. The findings may also indicate that the Nutri-Score system may require more public education than more intuitive symbols such as the *high in* labels. Future research should compare the impacts of different FOP formats on purchasing of both targeted nutrients and broader outcomes related to overall diet quality and implications for health.

No interaction effects were observed between the tax and FOP labelling conditions. However, the findings demonstrate that taxation and FOP labels have independent effects, which remained in the presence of the other policy. In other words, FOP labels had an effect above and beyond the effects of taxation, and vice versa. The cumulative effects of the tax and label interventions were considerable, suggesting greater public health benefit when both policies are implemented.

Several limitations should be noted. First, the study did not use a systematic sampling method, limiting generalizability to the larger Canadian population. However, the sample provided a large age range and good variability across sociodemographic characteristics, with notable similarities to the Canadian population in the proportion of participants identifying as Indigenous [68]. This study used an experimental marketplace design to replicate authentic purchasing behaviours as closely as possible; however, it may not represent how consumers interact with price and labels in real world settings, in which other influences (e.g., family members’ or peers’ preferences) may come in to play. Additionally, participants did not make purchases with their own money, which may have lead to more carefree spending. Both policy measures tested in this study were presented to participants without an associated description or explanation. Only about half of the participants reported noticing the FOP labels when they were present, which is substantially lower than rates of consumer awareness in countries with existing mandatory FOP labelling systems [69–71]. Notably, over a quarter of the participants randomized to the control condition (who were shown no FOP labels) reported seeing ‘nutrition labels or symbols’, suggesting that even fewer of the other participants may have actually noticed the FOP labels of interest, even if they reported so. Therefore, effect sizes may be greater under real world conditions, in which consumers are more likely to be aware of a FOP labelling system. Strengths of the study include the use of a randomized between-within experimental design, and behavioural outcomes with ‘real’ monetary consequences. Indeed, few studies to date have combined the high internal validity provided by an experimental design with actual purchase tasks.

## Conclusions

The study findings provide empirical support for the effectiveness of sugar taxes and FOP nutrition labels to help reduce consumption of sugars, sodium, saturated fats, and calories. Results suggest that including 100% fruit juice in the scope of taxed beverages leads to greater reductions in sugar consumption, and that sugar taxes may help to reduce consumption of sodium and saturated fats in addition to sugars and calories. Among FOP label designs, nutrient-specific FOP ‘high in’ warnings produced the most consistent reductions in nutrients of concern, reinforcing the approach taken in Chile and regulatory proposals in Canada and Brazil. Further ‘post-implementation’ research is required to understand how such interventions, on their own and in combination, affect overall diet quality at the population level.

## Additional file

Additional file 1: **Figure S1.** Visual depiction of the purchasing tasks protocol in the experimental marketplace. **Table S1.** Ratings/labels corresponding to label conditions for all beverage and food products included in the purchasing tasks. **Table S2.** Prices corresponding to tax conditions for all beverage and food products included in the purchasing tasks. **Table S3.** Nutrition information of all beverage and food products included in the purchasing tasks. (PDF 544 kb)

## Abbreviations

BMI: Body mass index
FOP: Front-of-package
MTL: Multiple traffic light
SD: Sugary drink
SSB: Sugar-sweetened beverage
WHO: World Health Organization
