# Supplementary material for: Taxes and front-of-package labels improve the healthiness of beverage and snack purchases: a randomized experimental marketplace
Source: Int J Behav Nutr Phys Act. 2019 May 21;16:46. doi: 10.1186/s12966-019-0799-0 (PMC6528263; doi:10.1186/s12966-019-0799-0)
Supplement: Supplementary file 1 — Figure S1. Visual depiction of the purchasing tasks protocol in the experimental marketplace. Table S1. Ratings/labels corresponding to label conditions for all beverage and food products included in the purchasing tasks. Table S2. Prices corresponding to tax conditions for all beverage and food products included in the purchasing tasks. Table S3. Nutrition information of all beverage and food products included in the purchasing tasks. (PDF 544 kb) [file 12966_2019_799_MOESM1_ESM.pdf]

Additional file 1

CONTENTS:

Figure S1 – Visual depiction of the purchasing tasks protocol in the experimental marketplace………………………………………………………………………………………… 1

Table S1 – Ratings/labels corresponding to label conditions for all beverage and food products included in the purchasing tasks …………………………………….. 4

Table S2 – Prices corresponding to tax conditions for all beverage and food products included in the purchasing tasks ………………………………………………….. 2

Table S3 – Nutrition information of all beverage and food products included in the purchasing tasks ……………………………………………………………………………….. 6

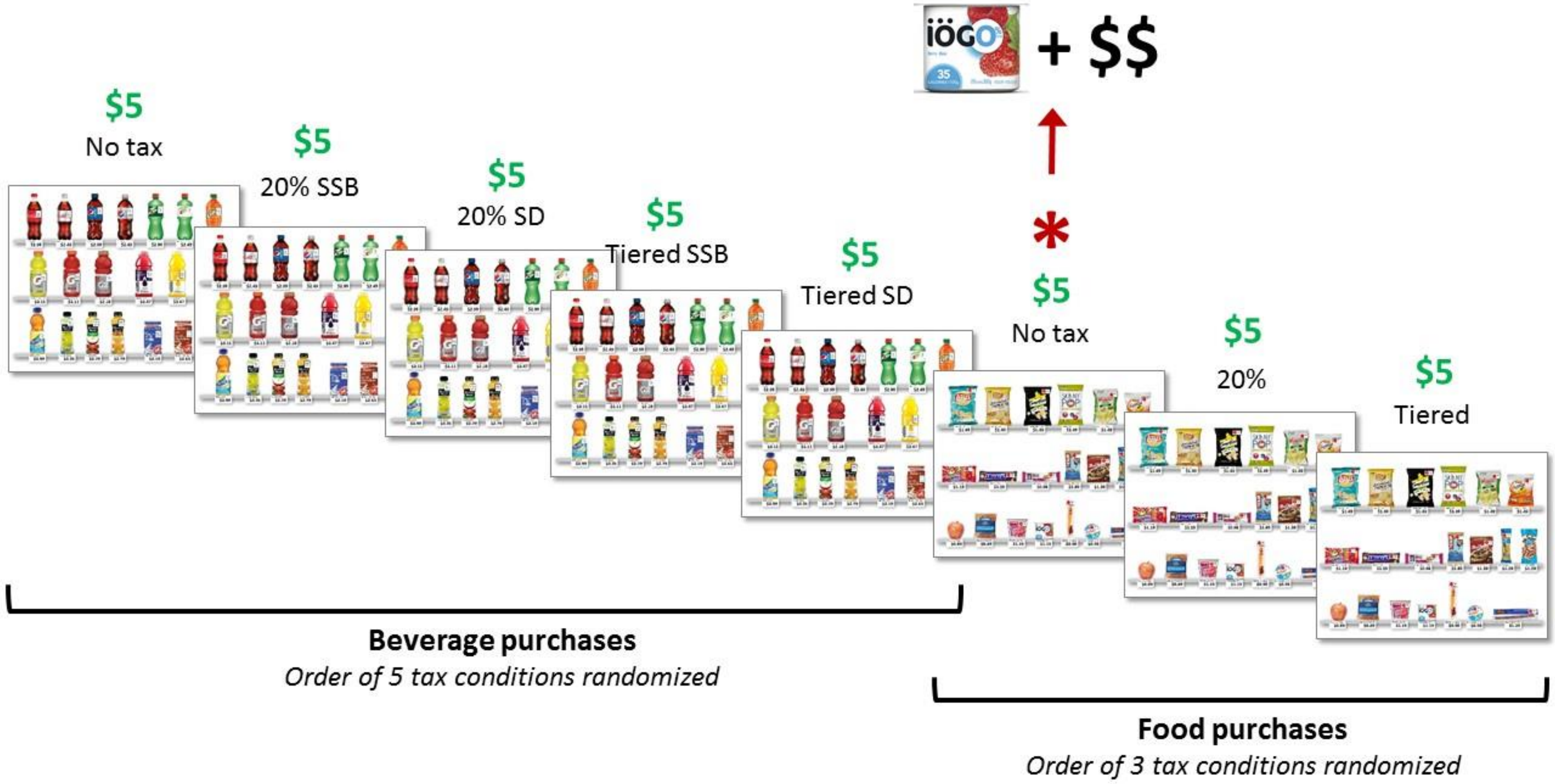

Figure S1. Visual depiction of the purchasing tasks protocol in the experimental marketplace. Each respondent completed 8 consecutive purchasing tasks, with one randomly assigned FOP labelling scheme applied to the products throughout all 8 tasks. The products available for purchase (first beverages, and then snack foods) were displayed to participants on large print-outs. A budget of $5 was provided for each task. *At the end of the experiment, the computer randomly selected one of the respondent's eight selections to be their real purchase, and the respondent was given the actual food or beverage item and any change from that task.

Table S1. Ratings/labels corresponding to label conditions for all beverage and food products included in the purchasing tasks

		High in			MTL			Health star rating	Nutrition grade
		Sugar	*Sodium*	*Sat fat*	*Sugar*	*Sodium*	*Sat fat*		
**Beverages**									
Product	*Flavour/variety*								
Coca Cola		●			High	Low	Low	★	E
Diet Coke					Low	Low	Low	★★	D
Pepsi		●			High	Low	Low	★	E
Diet Pepsi					Low	Low	Low	★★	D
7-Up		●			High	Low	Low	★	E
Diet 7-Up					Low	Low	Low	★★	D
Orange Crush		●			High	Low	Low	★	E
Gatorade Original	Lemon-Lime	●			High	Low	Low	★1/2	D
Gatorade Original	Fruit Punch	●			High	Low	Low	★1/2	D
Gatorade Low-Cal G2	Fruit Punch				Low	Low	Low	★★	D
VitaminWater	XXX (berry-pomegranate)	●			High	Low	Low	★1/2	D
VitaminWater	Energy (tropical citrus)	●			High	Low	Low	★1/2	D
VitaminWater ZERO	XOXOX (diet berry-pomegranate)				Low	Low	Low	★★	D
Nestea Lemon Iced Tea		●			High	Low	Low	★1/2	D
Minute Maid Lemonade		●			High	Low	Low	★1/2	D
Minute Maid Apple Juice		●			High	Low	Low	★★★★★	A
Minute Maid Orange Juice		●			High	Low	Low	★★★★★	A
Neilson 2% White Milk				●	Medium	Low	High	★★★★1/2	A
Neilson 1% Chocolate Milk		●			High	Low	Low	★★★★	B
Real Canadian Spring Water					Low	Low	Low	★★★★★	A
**Foods**									
Product	*Flavour/variety*								
Potato chips	Lay's Salt & Vinegar		●		Low	High	Medium	★★1/2	C
Potato chips	Lay's Oven Baked Original				Medium	Medium	Medium	★★★★	B
Popcorn	Smartfood White Cheddar		●	●	Low	High	High	★★	D

Popcorn	Skinny Pop				Low	Medium	Medium	★★★★	B
Crackers/snack	Garden Veggie Straws		●		Low	High	Medium	★★1/2	C
Crackers/snack	Cheddar Goldfish		●		Low	High	Medium	★★	D
Candy gummies	Maynards Gummy Bears	●			High	Low	Low	★★	D
Chocolate bar	Snickers	●		●	High	Medium	High	★	E
Granola/cereal bar	Quaker Chewy Yogurt Bars	●		●	High	Medium	High	★1/2	D
Power/energy bar	Clif Energy Bar Chocolate Chip	●			High	Medium	Medium	★★1/2	C
Cookies	Mrs. Fields	●		●	High	Medium	High	★	E
Nuts	Planters Salted Peanuts			●	Low	Medium	High	★★★★	B
Nuts	Blue Diamond Salted Almonds				Low	Medium	Medium	★★★★★	A
Fresh fruit	Apple	*N/A*	*N/A*	*N/A*	*N/A*	*N/A*	*N/A*	*N/A*	*N/A*
Fresh vegetable	Baby Carrots Snack Pack	*N/A*	*N/A*	*N/A*	*N/A*	*N/A*	*N/A*	*N/A*	*N/A*
Yogurt	Beatrice Strawberry Fruit Bottom	●		●	High	Low	High	★★1/2	C
Yogurt	Iögo Fat Free Berry				Low	Low	Low	★★★★1/2	A
Cheese snack	Marbelicious Cheestrings		●	●	Low	High	High	★★★★★	A
Cheese snack	Mini-Babybel, Light		●	●	Low	High	High	★★★★★	A
Meat snack	Schneiders Hot Rods		●	●	Medium	High	High	1/2	E

Table S2. Prices corresponding to tax conditions for all beverage and food products included in the purchasing tasks

		Tax Conditions				
		**No tax**	**20% SSB** [a]	**20% SD** [b]	**Tiered SSB** [c]	**Tiered SD** [d]
**Beverages**						
Product	*Flavour/variety*					
Coca Cola		$ 2.49	$ 2.99 *	$ 2.99 *	$ 2.99 *	$ 2.99 *
Diet Coke		$ 2.49	$ 2.49	$ 2.49	$ 2.49	$ 2.49
Pepsi		$ 2.49	$ 2.99 *	$ 2.99 *	$ 2.99 *	$ 2.99 *
Diet Pepsi		$ 2.49	$ 2.49	$ 2.49	$ 2.49	$ 2.49
7-Up		$ 2.49	$ 2.99 *	$ 2.99 *	$ 2.99 *	$ 2.99 *
Diet 7-Up		$ 2.49	$ 2.49	$ 2.49	$ 2.49	$ 2.49
Orange Crush		$ 2.49	$ 2.99 *	$ 2.99 *	$ 2.99 *	$ 2.99 *
Gatorade Original	Lemon-Lime	$ 2.59	$ 3.11 *	$ 3.11 *	$ 2.85 *	$ 2.85 *
Gatorade Original	Fruit Punch	$ 2.59	$ 3.11 *	$ 3.11 *	$ 2.85 *	$ 2.85 *
Gatorade Low-Cal G2	Fruit Punch	$ 2.59	$ 2.59	$ 2.59	$ 2.59	$ 2.59
VitaminWater	XXX (berry-pomegranate)	$ 2.89	$ 3.47 *	$ 3.47 *	$ 3.18 *	$ 3.18 *
VitaminWater	Energy (tropical citrus)	$ 2.89	$ 3.47 *	$ 3.47 *	$ 3.18 *	$ 3.18 *
VitaminWater ZERO	XOXOX (diet berry-pomegranate)	$ 2.89	$ 2.89	$ 2.89	$ 2.89	$ 2.89
Nestea Lemon Iced Tea		$ 2.49	$ 2.99 *	$ 2.99 *	$ 2.99 *	$ 2.99 *
Minute Maid Lemonade		$ 2.79	$ 3.35 *	$ 3.35 *	$ 3.35 *	$ 3.35 *
Minute Maid Apple Juice		$ 2.79	$ 2.79	$ 3.35 *	$ 2.79	$ 3.35 *
Minute Maid Orange Juice		$ 2.79	$ 2.79	$ 3.35 *	$ 2.79	$ 3.35 *
Neilson 2% White Milk		$ 2.19	$ 2.19	$ 2.19	$ 2.19	$ 2.19
Neilson 1% Chocolate Milk		$ 2.19	$ 2.63 *	$ 2.63 *	$ 2.63 *	$ 2.63 *
Real Canadian Spring Water		$ 1.69	$ 1.69	$ 1.69	$ 1.69	$ 1.69
**Foods**						
Product	*Flavour/variety*	**No tax**	**20%**		**Tiered**	
Potato chips	Lay's Salt & Vinegar	$ 1.49	$ 1.49		$ 1.49	
Potato chips	Lay's Oven Baked Original	$ 1.49	$ 1.49		$ 1.49	
Popcorn	Smartfood White Cheddar	$ 1.49	$ 1.49		$ 1.49	

Popcorn	Skinny Pop	$ 1.49	$ 1.49	$ 1.49
Crackers/snack	Garden Veggie Straws	$ 1.49	$ 1.49	$ 1.49
Crackers/snack	Cheddar Goldfish	$ 1.49	$ 1.49	$ 1.49
Candy gummies	Maynards Gummy Bears	$ 1.19	$ 1.43 *	$ 1.43 *
Chocolate bar	Snickers	$ 1.19	$ 1.43 *	$ 1.43 *
Granola/cereal bar	Quaker Chewy Yogurt Bars	$ 0.98	$ 1.18 *	$ 1.18 *
Power/energy bar	Clif Energy Bar Chocolate Chip	$ 1.89	$ 2.27 *	$ 2.27 *
Cookies	Mrs. Fields	$ 1.39	$ 1.67 *	$ 1.67 *
Nuts	Planters Salted Peanuts	$ 1.29	$ 1.29	$ 1.29
Nuts	Blue Diamond Salted Almonds	$ 1.29	$ 1.29	$ 1.29
Fresh fruit	Apple	$ 0.89	$ 0.89	$ 0.89
Fresh vegetable	Baby Carrots Snack Pack	$ 0.89	$ 0.89	$ 0.89
Yogurt	Beatrice Strawberry Fruit Bottom	$ 1.19	$ 1.43 *	$ 1.31 *
Yogurt	Iögo Fat Free Berry	$ 1.19	$ 1.19	$ 1.19
Cheese snack	Marbelicious Cheestrings	$ 0.98	$ 0.98	$ 0.98
Cheese snack	Mini-Babybel, Light	$ 0.98	$ 0.98	$ 0.98
Meat snack	Schneiders Hot Rods	$ 1.29	$ 1.29	$ 1.29

[a] Beverages containing > 5 g of *added sugar* per 100 ml were assigned a 20% tax.
[b] Beverages containing > 5 g of *free sugar* per 100 ml were assigned a 20% tax.
[c] Beverages containing > 5 g *added sugar* per 100 ml were assigned a 10% tax; beverages containing > 8 g of *added sugar* per 100 ml were assigned a 20% tax.
[d] Beverages containing > 5 g *free sugar* per 100 ml were assigned a 10% tax; beverages containing > 8 g of *free sugar* per 100 ml were assigned a 20% tax.
[e] Foods containing > 10 g total sugar per 100 g were assigned a 20% tax.
[f] Foods containing > 10 g total sugar per 100 g were assigned a 10% tax; foods containing > 20 g total sugar per 100 g were assigned a 20% tax.
* Tax applies.

Table S3. Nutrition information of all beverage and food products included in the purchasing tasks

		**Serving volume** (mL)	**Calories** (kcal)	**Sugar** (g)	**Sodium** (mg)	**Saturated fat** (g)
**Beverages**						
Product	*Flavour/variety*					
Coca Cola		500	200	55	40	0
Diet Coke		500	0	0	55	0
Pepsi		591	260	69	20	0
Diet Pepsi		591	0	0	40	0
7-Up		591	260	70	100	0
Diet 7-Up		591	5	0	100	0
Orange Crush		591	270	71	120	0
Gatorade Original	Lemon-Lime	591	150	35	250	0
Gatorade Original	Fruit Punch	591	150	35	250	0
Gatorade Low-Cal G2	Fruit Punch	591	50	12	270	0
VitaminWater	XXX (berry-pomegranate)	591	130	32	0	0
VitaminWater	Energy (tropical citrus)	591	120	32	0	0
VitaminWater ZERO	XOXOX (diet berry-pomegranate)	591	0	1	0	0
Nestea Lemon Iced Tea		500	160	43	50	0
Minute Maid Lemonade		450	200	52	30	0
Minute Maid Apple Juice		450	210	48	40	0
Minute Maid Orange Juice		450	220	45	30	0
Neilson 2% White Milk		250	130	12	120	3
Neilson 1% Chocolate Milk		250	160	26	170	2
Real Canadian Spring Water		500	0	0	0	0
**Foods**						
Product	*Flavour/variety*	(g)				
Potato chips	Lay's Salt & Vinegar	60	320	1	530	2
Potato chips	Lay's Oven Baked Original	32	150	3	180	0.5
Popcorn	Smartfood White Cheddar	45	250	2	370	3
Popcorn	Skinny Pop	18	100	0	45	0.5

Crackers/snack	Garden Veggie Straws	28	130	1	210	1
Crackers/snack	Cheddar Goldfish	28	130	0	230	1
Candy gummies	Maynards Gummy Bears	60	200	32	35	0
Chocolate bar	Snickers	47	220	24	115	4
Granola/cereal bar	Quaker Chewy Yogurt Bars	35	150	11	115	2.5
Power/energy bar	Clif Energy Bar Chocolate Chip	68	250	21	150	1.5
Cookies	Mrs. Fields	60	270	24	230	5
Nuts	Planters Salted Peanuts	60	390	3	180	6
Nuts	Blue Diamond Salted Almonds	23	140	1	70	1
Fresh fruit	Apple	150	80	16	0	0
Fresh vegetable	Baby Carrots Snack Pack	65	25	4	30	0
Yogurt	Beatrice Strawberry Fruit Bottom	175	170	25	100	3
Yogurt	Iögo Fat Free Berry	100	35	3	45	0
Cheese snack	Marbelicious Cheestrings	21	60	0	150	2
Cheese snack	Mini-Babybel, Light	20	45	0	140	1.5
Meat snack	Schneiders Hot Rods	19	105	1	375	4
